# Assessing the Impact of the National Smoking Ban in Indoor Public Places in China: Evidence from Quit Smoking Related Online Searches

**DOI:** 10.1371/journal.pone.0065577

**Published:** 2013-06-11

**Authors:** Jidong Huang, Rong Zheng, Sherry Emery

**Affiliations:** 1 Institute for Health Research and Policy, University of Illinois at Chicago, Chicago, Illinois, United States of America; 2 School of International Trade and Economics, University of International Business and Economics, Beijing, China; The University of Auckland, New Zealand

## Abstract

**Background:**

Despite the tremendous economic and health costs imposed on China by tobacco use, China lacks a proactive and systematic tobacco control surveillance and evaluation system, hampering research progress on tobacco-focused surveillance and evaluation studies.

**Methods:**

This paper uses online search query analyses to investigate changes in online search behavior among Chinese Internet users in response to the adoption of the national indoor public place smoking ban. Baidu Index and Google Trends were used to examine the volume of search queries containing three key search terms “Smoking Ban(s),” “Quit Smoking,” and “Electronic Cigarette(s),” along with the news coverage on the smoking ban, for the period 2009–2011.

**Findings:**

Our results show that the announcement and adoption of the indoor public place smoking ban in China generated significant increases in news coverage on smoking bans. There was a strong positive correlation between the media coverage of smoking bans and the volume of “Smoking Ban(s)” and “Quit Smoking” related search queries. The volume of search queries related to “Electronic Cigarette(s)” was also correlated with the smoking ban news coverage.

**Interpretation:**

To the extent it altered smoking-related online searches, our analyses suggest that the smoking ban had a significant effect, at least in the short run, on Chinese Internet users’ smoking-related behaviors. This research introduces a novel analytic tool, which could serve as an alternative tobacco control evaluation and behavior surveillance tool in the absence of timely or comprehensive population surveillance system. This research also highlights the importance of a comprehensive approach to tobacco control in China.

## Introduction

China bears one of the highest tobacco burdens in the world. The 2010 Global Adult Tobacco Survey estimated that 53% of men and 2.4% of women, or approximately 300 million adults, smoke in China [1], and more than 70% of Chinese nonsmokers are exposed to secondhand smoke [2]. The implementation of WHO’s Framework Convention on Tobacco Control (FCTC) in China could lead to a significant reduction in tobacco-related morbidity and mortality, both in China and globally [3], [4].

Since China ratified FCTC in 2006, the Chinese government has adopted and strengthened several tobacco control policies related to its commitment to FCTC [5], [6]. In a widely publicized press conference in May 2010, the Chinese Ministry of Health (CMH) announced that China would adopt a smoking ban in indoor public places in January 2011 [7], the five year mark of China’s ratification of FCTC. In reality, the smoking ban was not adopted until May 1, 2011 when CMH issued a formal decision to prohibit smoking in 28 indoor public places listed in the State Council Regulations [8].

To date, rigorous evaluation of the impact of China’s implementation of tobacco control policies has been limited. China lacks a proactive, comprehensive tobacco control surveillance and evaluation system, a key component of WHO’s MPOWER package [9]. Annual representative individual or household-level surveys of tobacco-related attitudes, beliefs and behaviors are largely unavailable, and there are considerable time lags between available data sources and the rapidly-changing landscape of tobacco use and tobacco control in China. Consequently, it is challenging to monitor and evaluate the impact of tobacco control policies and tobacco industry marketing and promotion on smoking-related attitudes and behaviors in China, and to provide real-time evidence on the effectiveness of various policy tools.

This paper uses online search query data, submitted by millions of Chinese Internet users daily, to examine changes in tobacco-related attitudes and behaviors. Online search queries are a uniquely valuable source of historical and real-time information, which have been used to identify timely temporal and spatial health behavioral trends, to predict public health events, and to evaluate the effectiveness of public health practices and policies [10]–[21].

In June 2012, China had 538 million Internet users, 80% of whom reported that using a search engine to obtain information (about products, services, employment, health care, etc) was one of their most frequent online activities (Instant messaging was the activity that Chinese Internet users were most likely to engage in while they were online, with 82.8% of users reported using instant messaging while online) [22]. In December 2009 alone, more than 13 billion search queries were conducted in China [23]. As the Internet penetration rate increases in China over time, these search query data could transform tobacco-focused studies and health behavior-related research in general.

According to the China Internet Network Information Center’s (CNNIC) annual search engine research reports, Baidu and Google were the top online search engines in mainland China between 2009 and 2011. Prior to Google’s 2010 exit from China, 90% of Chinese Internet users identified Baidu (77.2% in 2009; 78.7% in 2010) and Google (12.7% in 2009; 11.4% in 2010) as their first choice search engine. In 2011, more than 95% of Chinese users reported Baidu as their first choice online search engine, and only 1.8% identified Google as their first choice. Measured by search volume (as opposed to number of users), in October 2011, 78% of all online search queries in China were conducted on Baidu, and 2.6% were done in Google [23], [24]. This paper analyzes the volume of online news coverage of the smoking ban, as well as the volume of both Google and Baidu search queries that used phrases related to “Quit smoking’ and “Electronic Cigarettes” in China, before and after the smoking ban was adopted.

## Methods

Data on the count of news items containing information about “Smoking Ban(s)” were obtained from the Baidu News database. This database was compiled using automatic news-gathering robot programs that collect approximately 120,000 to 130,000 articles per day from more than 1,000 most-visited Chinese websites, including national and local news websites and networks for government health departments, health care organizations, and traditional media companies. More details on Baidu News database can be found at http://www.baidu.com/search/news_help.html.

To examine the impact of the adoption of the smoking ban on quit smoking related online search queries, we analyzed three Chinese search terms “禁烟(Smoking Ban(s)),” “戒烟(Quit Smoking/Stop Smoking/Smoking Cessation),” and “电子烟/电子香烟(Electronic Cigarette(s))” (Singular and plural are the same in Chinese) for the period from Jan 1 2009 to December 31 2011. Search queries related to “禁烟(Smoking Ban(s))” capture the overall interests in the smoking ban related news and information. “戒烟(Quit Smoking/Stop Smoking/Smoking Cessation)” encompasses a wide range of search queries related to smoking cessation methods and products, and provides indicative evidence on quit intentions and quit attempts among Chinese smokers who use Internet search engine to obtain quit smoking related information, and on the general interests in smoking cessation related information among non-smoking Internet users. Electronic nicotine delivery systems (also known as electronic cigarettes), are battery-operated products, which first emerged in China in 2004; they are designed to deliver nicotine, as well as flavor and other chemicals [25]. Unlike other nicotine-containing medications, such as transdermal patches, gum, inhalers and nasal sprays, all of which have demonstrated safety and efficacy in randomized controlled trials [26], data on the safety and efficacy of electronic cigarettes are very limited [27]. Despite the lack of scientific evidence, electronic cigarettes have been promoted as a safer and healthier alternative to cigarettes [28], which enables users to get around the restrictions of smoking bans.

In this study, we rely on “电子烟/电子香烟(Electronic Cigarette(s))” related queries to capture smokers’ intentions or attempts to engage in compensatory behaviors after the smoking ban was implemented, as well as non-smokers’ electronic cigarette information seeking for themselves or on behalf of their family members or friends who are smokers. While the compensatory behaviors in response to the smoking ban may include other actions, such as use of smokeless tobacco, we choose to focus on electronic cigarettes because of the rapid growth of electronic cigarettes in recent years [28] and also because very few Chinese adults use smokeless tobacco products (prevalence of current use of smokeless tobacco is 0.9% among male adults and 0.1% among female adults in 2010) [29].

The three search terms were specified in simplified Chinese characters (Traditional Chinese characters are used mainly in Taiwan and Hong Kong). Search query data were restricted to mainland China in both Baidu Index and Google Trends. Online search query data, along with the news coverage on the smoking ban, were accessed from Baidu.com and Google.com using Baidu Index (http://index.baidu.com/) and Google Trends (http://www.google.com/trends/). The Google search query volume data used in this paper were obtained from Google Insights for Search (http://www.google.com/insights/search/), launched by Google in 2008. In September 2012, Google Insights for Search was redesigned and merged with Google Trends (http://www.google.com/trends/), which was launched in 2007 (http://insidesearch.blogspot.co.il/2012/09/insights-into-what-world-is-searching.html).

Google Trends provides detailed daily or weekly data, depending on the search volume, for worldwide and local search volumes. Queries of search volume data can be customized by region and period. Google Trends provides relative search volume (RSV) for a given search term, indicating the number of searches for that term, relative to the total number of Google searches in a given region and over a user-specified time period. The Google Trends RSV represents a normalized value, scaled from 0–100; for example, RSV = 100 represents the highest search volume for a specified term, in a given region and time period, and RSV = 50 is 50% of the highest volume for that term/region/time period.

Baidu Index provides search volume data generated from the search queries submitted on Baidu.com since 2006. The Baidu Index represents a normalized number of unique searches for a search keyword, relative to total search volume on Baidu on a given day. Meaningful comparisons can be conducted using the search volume numbers generated by the Baidu Index for different search keywords for the same time period. For example, the search volume number provided by Baidu Index data for “戒烟(Quit Smoking/Stop Smoking/Smoking Cessation)” was 945 on January 1, 2011, and was 2,518 for “电子烟/电子香烟(Electronic Cigarettes),” and 24,902 for “周杰伦(Jay Chou, one of the most popular singers in China)” on the same day. While those numbers do not represent the absolute search volume for these keywords, as they have been normalized, the relative search volume can be easily calculated. In this case, the search volume of “Electronic Cigarette(s)” was 2.7 times that of “Quiting Smoking” in Baidu on January 1, 2011, which was approximately one tenth of the search volume for “Jay Chou.”

Baidu Index also provides basic demographic (age group and gender), geographic (city and province where search queries are originated), and socio-economic (occupation and education) characteristics of users who submitted search queries related to a given search keyword. These data are based on login-users’ self-reported information and their IP addresses. Baidu also provides predicted characteristics for anonymous users (who are not logged in when they perform a search); predicted values are based on undisclosed data mining methods, and should be used with strong caution. Detailed description of Baidu Index can be found at http://www.baidu.com/search/index_help.html.

## Results

### Smoking Ban(s) News Articles and Smoking Ban(s) Related Searches

**Figure 1:**
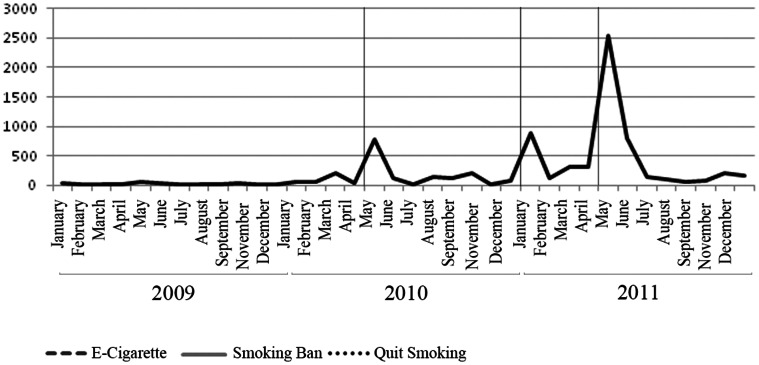
Number of News Items on Smoking Bans in Chinese Media. Marked Dates May 1, 2010: Smoking ban announced by CMOH January 1, 2011: Smoking ban effective date initially announced by CMOH May 1, 2011: Smoking ban adopted and became effective.

Figure 1 displays the monthly number of news articles related to “Smoking Ban(s)” in the Chinese news media from January 2009 to December 2011. Prior to May 2010, there were very few “Smoking Ban(s)” news articles. The number of such articles first spiked in May 2010, corresponding to the CMH’s high profile press conference, and subsequently dropped; the new level remained higher than the pre-2010 May period. The second spike came in January 2011, the five year mark of China’s ratification of FCTC, at which time China was expected to implement comprehensive indoor public place smoking bans per FCTC mandate. The largest monthly number of “Smoking Ban(s)” news articles (2,530), occurred in May 2011 when CMH issued a formal decision to prohibit smoking in 28 indoor public places listed in the State Council Regulations.

**Figure 2:**
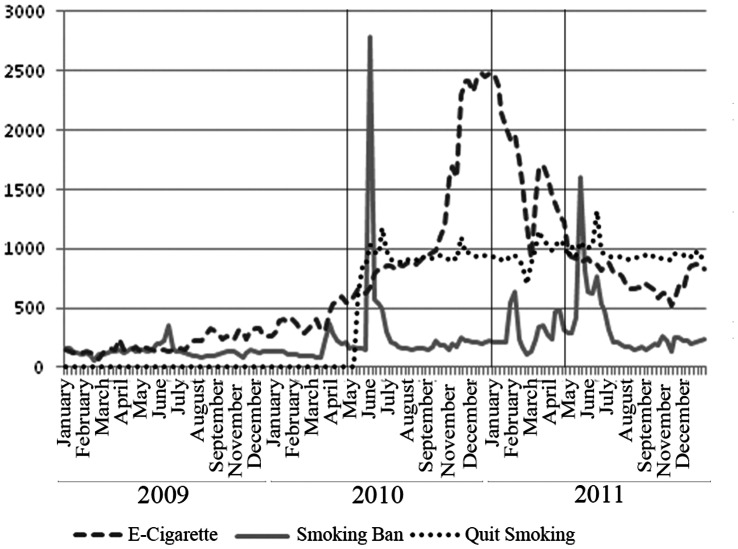
Baidu Relative Search Volume. Marked Dates May 1, 2010: Smoking ban announced by CMOH January 1, 2011: Smoking ban effective date initially announced by CMOH May 1, 2011: Smoking ban adopted and became effective.

**Figure 3:**
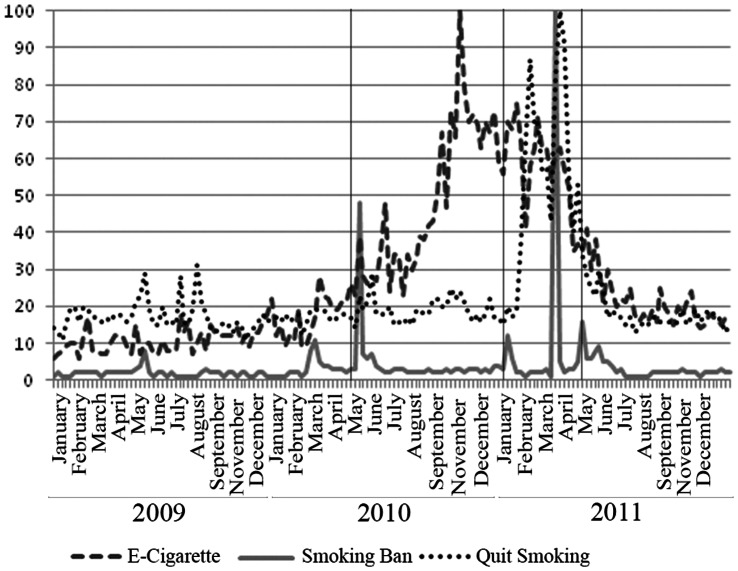
Google Relative Search Volume. Marked Dates May 1, 2010: Smoking ban announced by CMOH January 1, 2011: Smoking ban effective date initially announced by CMOH May 1, 2011: Smoking ban adopted and became effective.

Chinese Internet users’ interest in and awareness of the smoking ban was evident from the Baidu Index search query volume data for “禁烟(Smoking Ban(s)),” illustrated in Figure 2 (the solid line), and from Google Trends data in Figure 3 (the solid line). The pattern of Baidu search query volume on “Smoking Ban(s)” closely follows the pattern of the monthly number of “Smoking Ban(s)” news articles in Figure 1. Prior to May 2010, there were few “Smoking Ban(s)” related searches among Baidu users. Such searches climbed sharply immediately following the CMH’s May 2010 press conference. The search query volume dropped subsequently, but spiked again around February and May 2011, corresponding to the pattern in the number of “Smoking Ban(s)” news articles. Statistical analysis revealed a positive and statistically significant (p<0.01) correlation between the Baidu search query volume and “Smoking Ban(s)” news articles. Similar spikes were observed in the search query volume data from Google Trends, with the highest RSV in the weeks between late March and early April of 2011, a time when the smoking ban bill was intensely discussed during China’s annual legislative sessions.

### Quit Smoking Related Searches

In addition to showing the volume of search queries related to the smoking ban, Figures 2 (Baidu) and 3 (Google) also illustrate the volume of search queries containing the keywords “戒烟(Quit Smoking/Stop Smoking/Smoking Cessation)” and “电子烟/电子香烟(Electronic Cigarette(s)).” Similar to the pattern for “Smoking Ban(s),” Baidu search volume for “Quit Smoking” surged immediately following the May 2010 CMH press conference, from close to zero to approximately 1,000 units of standardized searches/day (As discussed in the Method section, Baidu search query volume measure is normalized. The actual search volume corresponds to one standardized unit is not disclosed). That level was sustained, with minor fluctuations, and surged again in late June and early July of 2011, about one month after the smoking ban was adopted.

These search query volume data show a clear, albeit slightly lagged, positive correlation between searches for “Smoking Ban(s)” and “Quit Smoking.” This pattern suggests an increased and sustained interest in quit smoking related information among Baidu users corresponding to the announcement and the final adoption of the smoking ban. The data from Google Trends also show the highest volume of “Quit Smoking” related search queries occurred in February and April of 2011, coinciding with the surges in search volume for “Smoking Ban(s)”on Google. The Google Trends data also show a strong and slightly lagged positive correlation between the volume of “Smoking Ban(s)” related searches and “Quit Smoking” related searches.

Table 1 summarizes the demographic and socio-economic (SES) profiles of those who submitted “Quit Smoking” related search queries on Baidu. The characteristics of user profiles were analyzed over three distinct time periods: pre-CMH press conference (Jan 2009 to April 2010), the announcement and adoption of the smoking ban (May 2010– May 2011), and post-adoption period (June 2011– Dec 2011). Table 1 shows that across all three time periods, “Quit Smoking” related search queries were largely submitted by those age 20–49 (84%), male (76%), with at least a high school degree (75%), and worked in IT, education, government and public services, and finance and real estate (72%), groups that are more likely to have access to and have higher rates of utilization of the Internet and smart phones [22]. The consistency in user characteristics suggests that the increase in the search volume was unlikely to have been driven by changes in Baidu users over this time period, but rather reflects a genuine increase of interest in quit smoking related information, prompted by news/information about the smoking ban. Geographically, the vast majority of “Quit Smoking” search queries came from cities and provinces in the east coastal area where the local economy is more developed, and individuals have greater access to and utilization of the Internet and smart phones. In addition, the local economies of these areas do not rely disproportionately on tobacco production and consumption.

**Table 1:** Baidu Quitting Smoking Searchers – Demographic Characteristics.

	1 Jan 2009 to 30-Apr-10	1 May 2010 to 31 May 2011	1 June 2011 to 31 Dec 2011
**Age (in years)**			
10–19	12%	12%	11%
20–29	27%	26%	27%
30–39	40%	40%	40%
40–49	18%	18%	18%
50–59	4%	4%	3%
**Sex**			
Male	76%	76%	76%
Female	24%	24%	24%
**Occupation**			
IT	30%	30%	31%
Education/Student	25%	25%	25%
Government/Public Service	9%	10%	10%
Finance/Real Estate	8%	9%	7%
Architecture	7%	6%	6%
Telecom/Internet	6%	5%	6%
Services	5%	5%	5%
Media/Entertainment	5%	4%	5%
Energy/Mining	3%	3%	3%
Retail	3%	3%	3%
**Education**			
College and above	28%	28%	29%
Professional school	19%	19%	18%
High school	28%	28%	28%
Middle school	13%	13%	13%
Primary school	13%	13%	12%

Table 2 presents the quarterly top-searched terms related to “Quit Smoking” in Baidu (Table S1 lists these search terms in their original Chinese format). “Smoking Cessation Products”, “Smoking Cessation Methods” and “How to Quit Smoking” were consistently among the most-searched terms between July 2009 - December 2011, indicating those who submitted “Quit Smoking” related search queries were most interested in the methods and products that could help quit smoking.

**Table 2:** Top searched terms related to “quit smoking” on baidu by quarter.

Rank	2009		2010				2011			
	Q3	Q4	Q1	Q2	Q3	Q4	Q1	Q2	Q3	Q4
1	smoking cessation products	smoking cessation products	smoking cessation methods	best ways to quit smoking	smoking cessation methods	smoking cessation products	smoking cessation methods	smoking cessation methods	smoking cessation methods	smoking cessation methods
2	smoking cessation methods	how to quit smoking	smoking cessation products	smoking cessation products	smoking cessation products	smoking cessation methods	smoking cessation products	how to quit smoking	how to quit smoking	how to quit smoking
3	how to quit smoking	smoking cessation methods	how to quit smoking	smoking cessation methods	how to quit smoking	how to quit smoking	how to quit smoking	the benefits of quit/stop smoking	quitting smoking bulletin boards system	quitting smoking bulletin boards system
4	the benefits ofquit/stop smoking	quitting smoking bulletin boards system	quit smoking folk medicine	smoking cessation methods	quitting smoking bulletin boards system	the benefits ofquit/stop smoking	quitting smoking bulletin boards system	quitting smoking bulletin boards system	the benefits ofquit/stop smoking	the benefits of quit/stop smoking
5	quitting smoking bulletin boards system	how to quit smoking	quitting smoking bulletin boards system	how to quit smoking	“Qing Fei Jie Yan Lin”™	quitting smoking bulletin boards system	the benefits ofquit/stop smoking	smoking cessation products	smoking cessation products	smoking cessation products
6	quit smoking folk medicine	the benefits ofquit/stop smoking	the benefits ofquit/stop smoking	quitting smoking bulletin boards system	the benefits ofquit/stop smoking	how to quit smoking	the easy way to stop smoking book	he easy way tostop smoking book	the easy way to stop smoking book	the easy way to stop smoking book
7	*	quit smoking folk medicine	how can one quit smoking	the benefits ofquit/stop smoking	how to quit smoking	“Qing Fei Jie Yan Lin”™	how to quit smoking	“Qing Fei Jie Yan Lin”™	“Qing Fei Jie Yan Lin”™	“Qing Fei Jie Yan Lin”™
8	*	*	how to quit smoking	quit smoking folk medicine	quit smoking folk medicine	quit smoking folk medicine	how to quit smoking	“Jie Yan Lin”™	“Jie Yan Lin”™	“Jie Yan Lin”™
9	*	*	smoking cessation forum/website	how to quit smoking for good	smoking cessation methods	“Jie Yan Lin”™	“Qing Fei Jie Yan Lin”™	smoking cessation toothpaste	smoking cessation toothpaste	smoking cessation toothpaste
10	*	*	best ways to quit smoking	persuade fathersto quit smoking	*	*	*	*	*	*

Note: The Chinese Characters “戒烟” can mean “Stop Smoking,” “Quit Smoking,” “Smoking Cessation,” and/or “No Tobacco.” The Chinese PinYin for “戒烟 is “Jie Yan.”.

The superscript “™” represents the trade mark or a brand name of a smoking cessation product.

The symbol “*” means the data are not available.

### Electronic Cigarettes Related Searches

Figures 2 and 3 illustrate search query volume related to electronic cigarettes. Baidu Index shows that “Electronic Cigarettes” related search query volume remained low, but gradually increased throughout 2009, accelerating in February 2010, ahead of the CMH’s press conference, and surging rapidly in the last quarter of 2010, and peaking in January 2011. Another spike in volume in March 2011 was followed by minor oscillations through the end of 2011. The surge in the last quarter of 2010 may have reflected anticipation of the adoption of smoking ban in January 2011, the time when CMH first announced the ban would take effect. However, this increase may also have resulted from industry marketing efforts prior to the New Year. Google search volume for “Electronic Cigarettes” related queries shows similar patterns, with the highest proportion of searches in the weeks between late October and early November of 2010, and small spikes around the three key events.

Compared to “Quit Smoking” related searches, the search volume for “Electronic Cigarettes” does not exhibit the same clear, positive relationship with the search volume for “Smoking Bans.” However, the Biadu search volume for “Electronic Cigarette(s)” accelerated ahead of the three key dates, and Google search volume for “Electronic Cigarette(s)” also spiked when the Google search volume for “Smoking Bans” increased.

Table 3 shows that, similar to the users who submitted “Quit Smoking” related search queries, “Electronic Cigarette(s)” related search queries were largely generated by male (78%) users, age 20–49 (87%), with at least a high school degree (79%), who worked in IT, education, government and public services, and finance and real estate (71%). There were no major shifts in the demographic and SES characteristics across three time periods, and the heaviest search volume for “Electronic Cigarettes” was concentrated in the east coastal area–Guang Dong province, in particular, which may be due to the fact that Guang Dong is the original manufacture and production base for electronic cigarettes in China and the world [25].

**Table 3:** Baidu E-cigarette Searchers – Demographic Characteristics.

Demographic Characteristics	1 Jan 2009–30 Apr 2010	1 May 2010–31 May 2011	1 June 2011–31 Dec 2011
**Age (in years)**			
10–19	10%	10%	8%
20–29	32%	33%	32%
30–39	43%	43%	45%
40–49	12%	10%	12%
50–59	3%	3%	3%
**Sex**			
Male	78%	78%	78%
Female	22%	22%	22%
**Occupation**			
IT	35%	36%	35%
Education/Student	22%	22%	23%
Government/Public Service	7%	9%	8%
Finance/Real Estate	7%	7%	6%
Telecom/Internet	6%	6%	6%
Services	6%	6%	6%
Architecture	5%	5%	5%
Media/Entertainment	5%	3%	5%
Retail	3%	3%	3%
Energy/Mining	3%	3%	3%
**Education**			
College and above	30%	29%	31%
Professional school	24%	25%	24%
High school	25%	24%	24%
Middle school	12%	11%	12%
Primary school	10%	10%	10%

Table 4 presented the most-searched terms related to “Electronic Cigarettes” on Baidu by quarter (Table S2 lists these search terms in their original Chinese format). “Electronic Cigarettes Health Risks,” “Electronic Cigarettes Brands,” and “Electronic Cigarettes Price” were among the top-searched terms between July 2009 and December 2011, indicating the Baidu users who submitted those queries were interested in purchasing electronic cigarettes as an alternative to traditional cigarettes, but also were concerned about their potential health risks.

**Table 4:** Top searched terms related to “electronic cigarette(s)” on baidu by quarter.

Rank	2009		2010				2011			
	Q3	Q4	Q1	Q2	Q3	Q4	Q1	Q2	Q3	Q4
1	“RUYAN”™ e-cigarette	“RUYAN”™ e-cigarette	e-cigarette price	healthy e-cigarettes	what are the health risks of e-cigarettes	what are the health risks of e-cigarettes	e-cigarette health risks	e-cigarette health risks	e-cigarette health risks	e-cigarette health risks
2	*	e-cigarette health risks	e-cigarette health risks	“RUYAN”™ e-cigarette	Do e-cigarettes work	Do e-cigarettes work	what are the health risks of e-cigarettes	e-cigarette brands	Do e-cigarettes work	“RUYAN”™ e-cigarette
3	*	e-cigarette price	“RUYAN”™ e-cigarette	e-cigarette price	healthy e-cigarettes	e-cigarette brands	e-cigarette brands	Do e-cigarettes work	e-cigarette brands	Do e-cigarettes work
4	*	*	healthy e-cigarettes	e-cigarette health risks	e-cigarette health risks	e-cigarette health risks	Do e-cigarettes work	“RUYAN”™ e-cigarette	“RUYAN”™ e-cigarette	e-cigarette brands
5	*	*	“Smoking Everywhere”™ e-cigarette	“Smoking Everywhere”™ e-cigarette	“RUYAN”™ e-cigarette	healthy e-cigarettes	“RUYAN”™ e-cigarette	e-cigarette price	e-cigarette price	e-cigarette price
6	*	*	*	*	e-cigarette price	e-cigarette price	e-cigarette price	healthy e-cigarettes	healthy e-cigarettes	healthy e-cigarettes
7	*	*	*	*	Do healthy e-cigarettes work	“RUYAN”™ e-cigarette	healthy e-cigarettes	*	*	*
8	*	*	*	*	Can e-cigarettes help quit smoking	what about e-cigarettes	what about e-cigarettes	*	*	*
9	*	*	*	*	what about e-cigarettes	Can e-cigarettes help quit smoking	best e-cigarettes	*	*	*
10	*	*	*	*	*	Do healthy e-cigarettes work	Can e-cigarettes help quit smoking	*	*	*

Note:

The superscript “™” represents the trade mark or a brand name of an electronic cigarette product.

The symbol “*” means the data are not available.

## Discussion and Conclusion

Despite the substantial economic and health costs imposed on China by tobacco use [4], [30]. China’s large population, vast geographic variations, and limited tobacco control resources make it extremely difficult to monitor and evaluate tobacco use, tobacco control policy and tobacco industry marketing and promotion strategies. Fortunately, the rapid diffusion of Internet use and users’ increasing reliance on search engines as a gateway to health related information make it increasingly feasible to conduct real-time tobacco surveillance and evaluation in China (China’s Internet penetration rate was 39.9% in June 2012, almost doubled since 2008, and was about one third higher than the world average. [22] According to a 2007 China Internet Network Information Center (CINIC) report, the Internet is the primary channel for obtaining information for Internet users (vs. Television for non-Internet users) [24]). This study illustrated the potential of using online search query data to conduct tobacco surveillance in China.

Our analyses show that the announcement and adoption of the indoor public place smoking ban in China generated significant increases in news coverage of smoking bans. Even after the smoking ban was adopted, the number of news articles related to smoking bans was higher than the pre-smoking ban period. Moreover, there was significant positive correlation between the number of news stories about smoking bans and the volume of “Smoking Ban(s)” and “Quit Smoking” related search queries. This pattern provides strong evidence that the announcement by CMH and subsequent adoption of the smoking ban prompted Chinese Internet users to search for “Smoking Ban(s)” and “Quit Smoking” related information online. Our results also provide some evidence that the announcement and adoption of the smoking ban may have contributed to the increase in “Electronic Cigarette(s)” related searches among the Chinese Internet users.

The vast majority of those Chinese Internet searches for “Smoking Ban(s),” “Quit Smoking” and “Electronic Cigarette(s)” came from users aged 20–49 (85%), male (77%), with at least a high school degree (76%), who worked in IT, education, government and public services, and finance and real estate related occupations (71%). While this finding may reflect those groups’ relatively greater access to and utilization of the Internet and search engines, the disproportionately high search volume coming from those groups related to “Quit Smoking” and “Electronic Cigarette(s)” cannot be fully explained by their greater online presence (A CINIC national survey on Chinese Internet users in early 2012 found that 55% of Chinese Internet users were male, 67.7% were between age 20–49, 53.5% had at least a high school degree, and 54.6% were in education, government and public services, management and professional occupations [22]). A second interpretation could be that men, aged 25–44, who have attended secondary school, have a greater likelihood of being a smoker, and hence may be more likely to search for smoking related information online. Interestingly, despite having much lower prevalence of smoking (2.4%), Chinese women contributed close to one quarter searches among all “Quit Smoking” and “Electronic Cigarette(s)” related searches, suggesting a strong interest in smoking related information among female Chinese Internet users.

This study has a few limitations. Mostly notably, the search query data left out some important population segments in China, particularly those who are older, having low-income or low level of education, or living in rural areas, where access to Internet is limited. Such disparities in Internet access and use will likely become less significant as economic development and technology advancement make information communication technologies cheaper, more user-friendly, and more accessible to a broader spectrum of Chinese society. In addition, although our key search term “戒烟(Quit Smoking/Stop Smoking/Smoking Cessation)” encompasses a wide range of smoking cessation methods and products related search queries, to the extent that some search queries do not contain the keyword “戒烟(Quit Smoking/Stop Smoking/Smoking Cessation),” we might leave out some important search queries, such as NRT products (patches, gum, lozenges, sprays, and inhalers) and oral medication. For example, a search query contains only “Bupropion” or “Nicotine gum” was not captured in our analysis. Our results are thus likely to underestimate the quit smoking related responses to the smoking ban. Future studies can improve our study by broadening search queries to include NRT products and oral medications, as well as methods such as acupuncture, herbs, aromatherapy, and hypnosis that may be used by Chinese smokers.

Despite these limitations, the results of this study demonstrated that search query data are a valuable resource for tobacco control efforts in China and can be used to monitor and evaluate smokers’ responses to tobacco control policy changes. Previous research has demonstrated that changes in smoking behavior among highly educated individuals prefigure broader changes across the general populations [31], [32]. To the extent changes in online search queries reflect changes in attitudes and behaviors among Chinese elite, they can also be considered a leading indicator of changes in smoking behavior among the general population in China. Moreover, given the correlation between search queries on electronic cigarettes and the smoking ban, our findings also pinpoint the importance of a comprehensive approach to tobacco control in China–encompassing novel tobacco products, as well as more traditional combustible tobacco. A recent study showed that media coverage can produce positive changes, or prevent negative changes in health-related behaviors across large populations [33]. Our results suggest that the impact of media coverage is likely moderated by the concurrent availability of cessation services and products. In the case of the smoking ban in China, the impact of the smoking ban and related news coverage may have been higher if they had been accompanied by other tobacco control policies, cessation support, and/or a media campaign that provided evidence-based cessation information.

Although our study demonstrated the potential of search query data as an alternative tobacco monitoring and evaluation tool in China, the stability and reliability of using search query data as a surveillance method need to be further assessed and tested in other settings. Since the passage of the national smoking ban, a number of provinces and municipalities in China have introduced stronger smoking bans in their jurisdictions. Future studies can assess the robustness of this method by examining whether smoking related search queries respond to those localized tobacco control policies, and whether these responses were specific to the localities in which these policies were adopted. In addition, future research can also test its reliability by examining the changes in the smoking related search queries in China when future national level tobacco control policies are adopted.

## Supporting Information

Table S1 Top Searched Terms Related to “Quit Smoking” on Baidu by Quarter (In Chinese).
(DOCX)

Table S2 Top Searched Terms Related to “Electronic Cigarette(s)” on Baidu by Quarter (In Chinese).
(DOCX)
