# Supplementary material for: Assessing the Impact of the National Smoking Ban in Indoor Public Places in China: Evidence from Quit Smoking Related Online Searches
Source: PLoS One. 2013 Jun 11;8(6):e65577. doi: 10.1371/journal.pone.0065577 (PMC3679166; doi:10.1371/journal.pone.0065577)
Supplement: Table S1 — Top Searched Terms Related to “Quit Smoking” on Baidu by Quarter (In Chinese). (DOCX) [file pone.0065577.s001.docx]

| **TABLE S1 TOP SEARCHED TERMS RELATED TO "QUIT SMOKING" ON BAIDU BY QUARTER (IN CHINESE)** | | | | | | | | | | |
| --- | --- | --- | --- | --- | --- | --- | --- | --- | --- | --- |
| Rank | 2009 | | 2010 | | | | 2011 | | | |
|  | Q3 | Q4 | Q1 | Q2 | Q3 | Q4 | Q1 | Q2 | Q3 | Q4 |
| 1 | 戒烟产品 | 戒烟产品 | 戒烟方法 | 戒烟的最好方法 | 戒烟的方法 | 戒烟产品 | 戒烟的方法 | 戒烟的方法 | 戒烟的方法 | 戒烟的方法 |
| 2 | 戒烟方法 | 如何戒烟 | 戒烟产品 | 戒烟产品 | 戒烟产品 | 戒烟的方法 | 戒烟产品 | 如何戒烟 | 如何戒烟 | 如何戒烟 |
| 3 | 如何戒烟 | 戒烟方法 | 如何戒烟 | 戒烟方法 | 如何戒烟 | 如何戒烟 | 如何戒烟 | 戒烟的好处 | 戒烟吧 | 戒烟吧 |
| 4 | 戒烟的好处 | 戒烟吧 | 戒烟偏方 | 戒烟的方法 | 戒烟吧 | 戒烟的好处 | 戒烟吧 | 戒烟吧 | 戒烟的好处 | 戒烟的好处 |
| 5 | 戒烟吧 | 怎么戒烟 | 戒烟吧 | 如何戒烟 | 清肺戒烟灵 | 戒烟吧 | 戒烟的好处 | 戒烟产品 | 戒烟产品 | 戒烟产品 |
| 6 | 戒烟偏方 | 戒烟的好处 | 戒烟的好处 | 戒烟吧 | 戒烟的好处 | 怎么戒烟 | 这书能让你戒烟 | 这书能让你戒烟 | 这书能让你戒烟 | 这书能让你戒烟 |
| 7 | 我要为你戒烟 | 戒烟偏方 | 怎么戒烟 | 戒烟的好处 | 怎么戒烟 | 清肺戒烟灵 | 怎么戒烟 | 清肺戒烟灵 | 清肺戒烟灵 | 清肺戒烟灵 |
| 8 | * | * | 怎样戒烟 | 戒烟偏方 | 戒烟偏方 | 戒烟偏方 | 怎样戒烟 | 戒烟灵 | 戒烟灵 | 戒烟灵 |
| 9 | * | * | 戒烟网 | 如何有效戒烟 | 戒烟方法 | 戒烟灵 | 清肺戒烟灵 | 戒烟牙膏 | 戒烟牙膏 | 戒烟牙膏 |
| 10 | * | * | 戒烟的最好方法 | 劝说爸爸戒烟 | * | * | * | * | * | * |
| Note: The symbol "*" means the data are not available. | | | | | | | | | | |
