# Supplementary material for: Assessing the Impact of the National Smoking Ban in Indoor Public Places in China: Evidence from Quit Smoking Related Online Searches
Source: PLoS One. 2013 Jun 11;8(6):e65577. doi: 10.1371/journal.pone.0065577 (PMC3679166; doi:10.1371/journal.pone.0065577)
Supplement: Table S2 — Top Searched Terms Related to “Electronic Cigarette(s)” on Baidu by Quarter (In Chinese). (DOCX) [file pone.0065577.s002.docx]

| **TABLE S2 TOP SEARCHED TERMS RELATED TO "ELECTRONIC CIGARETTE(S)" ON BAIDU BY QUARTER (IN CHINESE)** | | | | | | | | | | |
| --- | --- | --- | --- | --- | --- | --- | --- | --- | --- | --- |
| Rank | 2009 | | 2010 | | | | 2011 | | | |
|  | Q3 | Q4 | Q1 | Q2 | Q3 | Q4 | Q1 | Q2 | Q3 | Q4 |
| 1 | 如烟电子烟 | 如烟电子烟 | 电子烟价格 | 健康电子烟 | 电子烟有什么危害 | 电子烟有什么危害 | 电子烟的危害 | 电子烟的危害 | 电子烟的危害 | 电子烟的危害 |
| 2 | * | 电子烟的危害 | 电子烟的危害 | 如烟电子烟 | 电子烟有用吗 | 电子烟有用吗 | 电子烟有什么危害 | 电子烟品牌 | 电子烟有用吗 | 如烟电子烟 |
| 3 | * | 电子烟价格 | 如烟电子烟 | 电子烟价格 | 健康电子烟 | 电子烟品牌 | 电子烟品牌 | 电子烟有用吗 | 电子烟品牌 | 电子烟有用吗 |
| 4 | * | * | 健康电子烟 | 电子烟的危害 | 电子烟的危害 | 电子烟的危害 | 电子烟有用吗 | 如烟电子烟 | 如烟电子烟 | 电子烟品牌 |
| 5 | * | * | 益品电子烟 | 益品电子烟 | 如烟电子烟 | 健康电子烟 | 如烟电子烟 | 电子烟价格 | 电子烟价格 | 电子烟价格 |
| 6 | * | * | * | * | 电子烟价格 | 电子烟价格 | 电子烟价格 | 健康电子烟 | 健康电子烟 | 健康电子烟 |
| 7 | * | * | * | * | 健康电子烟有用吗 | 如烟电子烟 | 健康电子烟 | * | * | * |
| 8 | * | * | * | * | 电子烟能戒烟吗 | 电子烟怎么样 | 电子烟怎么样 | * | * | * |
| 9 | * | * | * | * | 电子烟怎么样 | 电子烟能戒烟吗 | 最好的电子烟 | * | * | * |
| 10 | * | * | * | * | * | 健康电子烟有用吗 | 电子烟能戒烟吗 | * | * | * |
| Note:  The symbol "*" means the data are not available. | | | | | | | | | | |
